# Prostatic Derangement

**Published:** 1878-04

**Authors:** R. W. Westmoreland

**Affiliations:** Washington, Arkansas


### PROSTATIC DERANGEMENT.

By R. W. WESTMORELAND, M.D., Washington, Arkansas.

Hypertrophy of the prostate is an affection troublesome to young people as well as old. The question of its degenerative character, or of its being the result of exciting causes, has very great bearing in deciding upon treatment. There is no doubt but that the affection is from distinct causes, and the object mainly sought after in attempts to cure, should be the removal of these causes. Thus in lithiasis we may have a condition of the urine suited to the production of the irritation in the genito-urinary apparatus sufficient to excite the prostatic trouble. Forms of local congestion or inflammation may give rise to very serious prostatic derangement, as is evidenced in cases originating in excessive venery, suppression, under certain circumstances, of the dermoid function, and horseback riding.

It should ever be the practice, in treating these cases, to search out the origin, and instead of always "poking" at the seat of trouble, remove the cause, and but little more will be necessary to relieve the patient. Gross has thus advised in the treatment of cystic irritability, and the words are very appropriate to the subject of this paper:

"In entering upon the treatment of this complaint, so protean in its character, a strict inquiry should, in every instance, be instituted into its origin, which, as has been already seen, may be either sympathetic, nervous, congestive or inflammatory, and the practice regulated accordingly; otherwise, the physician will only be likely to harass his patient and employ means which can lead to no beneficial result."

The direct sympathy existing between the bladder and gland renders it a very difficult matter, at times, to decide which is primarily affected, and whether the one or the other is affected by some originating cause. Like in diseases of the liver and stomach, we are very often at a loss to decide whether sympathetic or primary disturbance exists in the one or the other. This is a difficulty of very little importance, however, since exciting causes affecting the one redound to the detriment of the other, and removing the cause will very often cure both without much further trouble.

It is in the aged, particularly, that we are most often called on to treat this difficulty. We will have them complain of a burning, scalding sensation while urinating. They are affected by a certain amount of strangury, or, at times, with incontinence. All these symptoms may alternate in the same patient, and be temporarily aggravated by measures or remedies having a direct exciting tendency on the genito-urinary apparatus. Thus the administration of cantharis, either internally or as a blister, has a very decided effect in that direction.

Then, too, you find many enthusiastic specialists, whose principal recommendation is their fervor, with the idea of "incipient stricture" fixed in their mind, "job" away at the congested and irritable neck, in their vain endeavor to find even a minute variance in the calibre of the canal. Such a case I once knew of a printer, who came under my notice, after being "poked" at by a "stricture specialist," when he had nothing more than enlarged prostate and cervical irritability.

The incidental symptoms, such as spasmodic stricture, etc., amount to very little, unless unusually prolonged, as ordinarily they are of temporary character. Rectal suppositories will ameliorate them until the grand object of treatment, removing the cause, has been accomplished.

The rule is, then, that we may always expect to find some exciting cause for the affection, and we should never pronounce idiopathic that which may be found to be due to a distinct and separate disturbance. Should we be led to believe that the acrid property of the urine resulting from lithiasis, the origin, alkaline saline cathartics, potassium iodide and carbonate with buchu and belladonna, would be the best agents at our command to combat the difficulty.

Local applications are very seldom necessary, and when they are used, nitrate of silver or carbolate of iodine are about as efficient as anything that can be applied.

There is nothing better, in my opinion, than a preparation of belladonna and buchu, in counteracting the irritability always existing in connection with enlarged prostrate. By effecting this end we, in many forms of the disease, limit its progress, and quell many of the troubles incident thereto.

